# Hydrogels as Promising Carriers for Ophthalmic Disease Treatment: A Comprehensive Review

**DOI:** 10.3390/gels12020105

**Published:** 2026-01-27

**Authors:** Wenxiang Zhu, Mingfang Xia, Yahui He, Qiuling Huang, Zhimin Liao, Xiaobo Wang, Xiaoyu Zhou, Xuanchu Duan

**Affiliations:** 1 Aier Academy of Ophthalmology, Central South University, Changsha 410083, China; wenxiang@hnu.edu.cn (W.Z.); xmf_1033@126.com (M.X.); qlhuangsinap@163.com (Q.H.); liao8220@foxmail.com (Z.L.); wxiaobo@csu.edu.cn (X.W.); 2 College of Materials Science and Engineering, Hunan University, Changsha 410082, China

**Keywords:** hydrogel, ophthalmic diseases, drug delivery, corneal repair, glaucoma, retinal disorders, dry eye disease

## Abstract

Ocular disorders such as keratitis, glaucoma, age-related macular degeneration (AMD), diabetic retinopathy (DR), and dry eye disease (DED) are highly prevalent worldwide and remain major causes of visual impairment and blindness. Conventional therapeutic approaches for ocular diseases, such as eye drops, surgery, and laser therapy, are frequently hampered by limited drug bioavailability, rapid clearance, and treatment-related complications, primarily due to the eye’s unique anatomical and physiological barriers. Hydrogels, characterized by their three-dimensional network structure, high water content, excellent biocompatibility, and tunable physicochemical properties, have emerged as promising platforms for ophthalmic drug delivery. This review summarizes the classification, fabrication strategies, and essential properties of hydrogels, and highlights recent advances in their application to ocular diseases, including keratitis management, corneal wound repair, intraocular pressure regulation and neuroprotection in glaucoma, sustained drug delivery for AMD and DR, vitreous substitutes for retinal detachment, and therapies for DED. In particular, we highlight recent advances in stimuli-responsive hydrogels that enable spatiotemporally controlled drug release in response to ocular cues such as temperature, pH, redox state, and enzyme activity, thereby enhancing therapeutic precision and efficacy. Furthermore, this review critically evaluates translational aspects, including long-term ocular safety, clinical feasibility, manufacturing scalability, and regulatory challenges, which are often underrepresented in existing reviews. By integrating material science, ocular pathology, and translational considerations, this review aims to provide a comprehensive framework for the rational design of next-generation hydrogel systems and to facilitate their clinical translation in ophthalmic therapy.

## 1. Introduction

Ophthalmic diseases constitute a major global public health challenge, leading to substantial impairment of visual function and quality of life while imposing a considerable societal and economic burden [1,2,3]. Among these conditions, myopia remains highly prevalent and continues to increase worldwide. Systematic reviews indicate that between 1990 and 2023, the overall prevalence of myopia among children and adolescents rose from approximately 24% to 36%, and it is projected that nearly 740 million individuals in this age group will be affected globally by 2050 [4]. Beyond compromising everyday visual performance, high myopia markedly elevates the risk of vision-threatening complications, including retinal detachment and myopic maculopathy [5,6,7]. Glaucoma is another leading cause of irreversible blindness globally, with primary open-angle glaucoma being the most common subtype. Predictive models suggest that the prevalence of primary open-angle glaucoma among individuals aged 40 years and older will increase from 2.8% in 2024 to 3.5% by 2060, with the number of affected individuals rising from approximately 80.5 million to 187 million [8]. Population aging and the growing prevalence of myopia are considered major contributors to this upward trend [9,10]. In addition, retinal disorders such as age-related macular degeneration, diabetic retinopathy, and retinal detachment pose significant threats to vision in older adults and the working-age population, often resulting in sudden and severe visual loss in advanced stages [11,12,13,14]. The high prevalence, progressive nature, and irreversible visual impairment associated with these ophthalmic diseases underscore an urgent clinical need for therapeutic strategies that are not only more effective, but also safer and more convenient for long-term management.

Clinical treatment of ophthalmic diseases mainly includes three common methods: medication, surgery, and laser therapy [15,16]. Medication is suitable for conditions such as glaucoma and ocular infections, acting through drugs like beta blockers and antibiotics [17,18]. However, affected by multiple ocular barriers, drug bioavailability is low, requiring high doses or frequent administration, which may easily cause systemic side effects [19,20]. Surgery is a key treatment for diseases such as cataracts and retinal detachment. Although its efficacy is definite, it is invasive, carrying risks such as infection and bleeding, with a long postoperative recovery period and high costs, leading to limited accessibility in some regions [21]. Laser therapy is widely used in scenarios such as glaucoma, diabetic retinopathy, and refractive error correction. Nevertheless, it may cause complications like dry eye syndrome and corneal opacity, its long-term effects on the eye have not been fully clarified, and some patients may experience disease recurrence [22,23,24].

**Figure 1:**
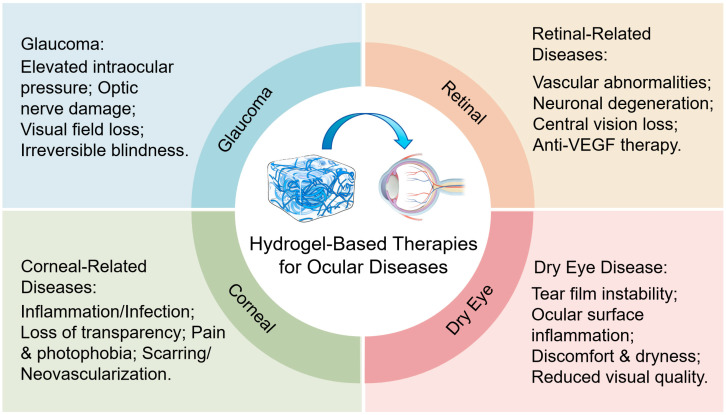
Schematic diagram of the application principle of hydrogels in the treatment of ophthalmic diseases.

As a highly promising drug delivery system in ophthalmic therapy, hydrogels offer significant advantages over traditional treatment methods. The three-dimensional network structure can encapsulate drugs, prevent degradation, and enable controlled release, thereby enhancing drug bioavailability, reducing dosage and systemic side effects [25,26,27]. By adhering to the ocular surface, hydrogels prolong drug retention time and decrease dosing frequency [28,29,30]. Additionally, hydrogels exhibit excellent biocompatibility, mimic the natural extracellular matrix properties of the eye, and reduce the risk of immune reactions and inflammation. Their mechanical properties can be customized as needed to adapt to the unique anatomical and physiological characteristics of the eye. Consequently, as shown in Figure 1, hydrogels effectively overcome the limitations of conventional treatments and hold great potential in the management of ophthalmic diseases [15]. Related research and applications are attracting increasingly widespread attention.

## 2. Hydrogels

### 2.1. Classification of Hydrogels

Hydrogel is a type of polymeric material characterized by a three-dimensional network structure. Its core property lies in the ability to absorb and retain large amounts of water while maintaining structural integrity and remaining insoluble [31,32,33]. Hydrogels can be classified into three categories based on their synthetic materials. Natural hydrogels are derived from natural polymers such as collagen and hyaluronic acid, offering excellent biocompatibility and biodegradability [34,35,36]. Collagen-based hydrogels have been widely used in tissue engineering. Synthetic hydrogels are made from synthetic polymers like polyethylene glycol (PEG), polyester (PLGA, PCL, etc.) and polyvinyl alcohol (PVA), allowing more flexible control over structure and properties, and can be functionalized to meet specific requirements [37,38,39]. Hybrid hydrogels combine the advantages of natural and synthetic polymers to achieve more comprehensive performance [40,41].

Based on cross-linking methods, hydrogels are divided into physical and chemical cross-linking types. Physically cross-linked hydrogels are formed through physical interactions such as hydrogen bonds and ionic bonds, exhibiting reversibility. Some polysaccharide-based hydrogels can undergo gel-sol transitions [42,43]. Chemically cross-linked hydrogels are formed via chemical reactions that create covalent bonds between polymer chains, resulting in greater mechanical stability and long-term structural integrity, though they are generally irreversible [44,45,46].

According to their response characteristics, hydrogels can be categorized as traditional or smart hydrogels. Traditional hydrogels maintain stable properties under conventional conditions and show no significant response to external stimuli. Smart hydrogels can perceive external stimuli such as temperature, pH, and light, and respond by altering physical and chemical properties like volume and permeability [47,48]. Examples include the temperature responsiveness of poly(N-isopropylacrylamide) (PNIPAM) hydrogels [49,50] and the pH responsiveness of hydrogels containing carboxyl or amino groups [51,52,53].

### 2.2. Intrinsic Relationship Between Hydrogel Fabrication Strategies and Performance Regulation

Different hydrogel fabrication methods not only determine the feasibility and operational conditions of the gelation process, but also profoundly influence the final physicochemical and biological properties by modulating network structural characteristics such as crosslinking density, pore size distribution, and dynamic reversibility. Therefore, in ophthalmic applications, the selection of a fabrication strategy is essentially a reverse design process aimed at achieving target performance.

For instance, chemical crosslinking methods form stable covalent bonds, which significantly enhance the mechanical strength and long-term structural stability of hydrogels [54]. This makes them suitable for scenarios requiring sustained mechanical stress or long-term implantation, such as vitreous substitutes or intraocular sustained-release implant systems. However, their typically higher crosslinking density often reduces swelling capacity and drug diffusion rates, necessitating a trade-off between mechanical performance and drug release efficiency during design [55,56].

In contrast, physically crosslinked hydrogels rely on hydrogen bonds, ionic interactions, or hydrophobic associations to form networks. These generally exhibit lower crosslinking energy and larger network pores, resulting in higher swelling ratios and faster drug release kinetics. Such hydrogels are prepared under mild conditions and demonstrate excellent biocompatibility, making them suitable for ocular surface drug delivery, corneal dressings, and in situ gelling eye drop systems [57,58]. However, their mechanical stability and long-term structural integrity are relatively limited [59].

Self-assembly and supramolecular crosslinking strategies construct dynamic networks through reversible non-covalent interactions, endowing hydrogels with injectability, self-healing capability, and stimuli-responsiveness [60]. These structural features offer unique advantages in applications that require adaptation to the dynamic ocular environment, on-demand drug release, or response to pathological signals—such as stimuli-responsive anti-inflammatory or neuroprotective hydrogels [61,62,63]. Nevertheless, the mechanical properties and long-term stability of such systems heavily depend on molecular design, which increases fabrication complexity and challenges in process scale-up [64,65].

Thus, the choice of fabrication method is not an isolated process decision but a key factor determining whether a hydrogel can simultaneously meet the requirements of mechanical compatibility, controlled drug release, biocompatibility, and clinical operability in specific ophthalmic applications. Establishing a clear correlation between fabrication strategies and target performance is crucial for advancing hydrogels from laboratory research to clinical translation.

### 2.3. Key Properties of Hydrogels for Ophthalmic Applications

The successful application of hydrogels in ophthalmic therapy critically depends on the precise matching of their physicochemical and biological properties with the unique physiological and pathological environments of the eye. Among these, biocompatibility, swelling behavior, mechanical properties, and environmental responsiveness are the key determinants governing safety, comfort, and therapeutic efficacy.

Biocompatibility and ocular tolerance represent the fundamental prerequisites for ophthalmic use. Hydrogels and their degradation products must not induce inflammatory responses, immune rejection, or cytotoxic effects in ocular tissues, including the cornea, conjunctiva, iris–ciliary body, or retina [66,67]. This requirement extends beyond material selection to encompass the entire fabrication process, including synthesis, purification, and sterilization. Residual monomers, crosslinkers, and organic solvents must be rigorously minimized to avoid potential impairment of visual function [68,69].

Swelling behavior plays a pivotal role in balancing drug release kinetics and patient comfort [70]. The equilibrium swelling ratio of hydrogels in tear fluid or aqueous humor directly influences network expansion and, consequently, drug diffusion rates and release duration. By modulating crosslinking density and hydrophilic–hydrophobic composition, drug release profiles can be programmed over time scales ranging from hours to several months. At the same time, swelling behavior strongly affects ocular comfort and visual performance. In applications such as ocular surface dressings or therapeutic contact lenses, excessive swelling may result in tightness, lens displacement, or visual blurring, whereas insufficient swelling compromises lubrication and moisture retention [71,72,73]. Therefore, ophthalmic hydrogels should be designed to maintain a moderate and stable swelling state under ocular conditions.

Mechanical properties must be quantitatively matched to the dynamic biomechanical environment of ocular tissues. The eye is continuously subjected to mechanical stimuli arising from blinking, ocular movements, and intraocular pressure fluctuations, requiring hydrogels to exhibit appropriate stiffness and resilience to ensure functional stability and safety [74,75]. In corneal applications, including corneal dressings, scaffolds, and therapeutic contact lenses, hydrogels should approximate the mechanical characteristics of the native corneal stroma. The elastic modulus of the human cornea is approximately 0.16–0.3 MPa [76]. Therefore, hydrogels for corneal applications are typically adjusted to a modulus range of 0.1–0.3 MPa to achieve a balance among mechanical support, wearing comfort, and tissue metabolism. Hydrogels with excessively low modulus are prone to collapse or displacement under shear stress induced by blinking, whereas overly stiff materials may cause foreign-body sensation, localized stress concentration, and disruption of corneal metabolism and transparency [77,78].

For vitreous substitutes and periretinal applications, mechanical matching becomes even more critical. Based on scientific literature, the vitreous modulus of pigs, cattle, sheep, and humans varies between 10^−2^~10^2^ Pa [79]. Hydrogels with moduli substantially exceeding this range may exert abnormal traction or compressive forces on the retina, increasing the risk of retinal damage or re-detachment, while materials with insufficient stiffness may fail to maintain intraocular structural integrity and physiological intraocular pressure. Consequently, vitreous-related hydrogels are generally designed to exhibit low moduli in the range of tens to several hundred pascals, while simultaneously maintaining high optical transparency, suitable rheological behavior, and long-term structural stability.

Stimuli-responsive properties further endow hydrogels with intelligent therapeutic potential. By incorporating environmental responsiveness, hydrogels can evolve from passive carriers into active systems capable of sensing and responding to disease-specific microenvironments [80]. For example, thermoresponsive hydrogels enable sol–gel transitions for in situ gelation following eye drop administration; pH-responsive hydrogels can target acidic microenvironments associated with infection or diabetic retinopathy; redox-responsive hydrogels allow triggered release of antioxidant agents in regions of elevated oxidative stress, such as diabetic retinopathy lesions; and enzyme-responsive hydrogels can be selectively activated by disease-associated overexpressed enzymes (e.g., matrix metalloproteinases), enabling more precise drug release [81,82,83,84]. Collectively, these features provide a materials-based foundation for the development of personalized and adaptive ophthalmic therapeutic strategies.

## 3. Application of Hydrogels in the Treatment of Ophthalmic Diseases

As shown in Table 1, our study systematically summarizes the application of hydrogels in the treatment of ophthalmic diseases in recent years.

### 3.1. Anterior Segment Diseases

#### 3.1.1. Keratitis

Keratitis is an inflammatory condition of the cornea that can be triggered by various factors, such as bacterial, viral, or fungal infections, as well as immune-mediated responses. If left untreated, it may lead to corneal scarring, ulcers, and even blindness [97,98]. Traditional treatments for keratitis primarily involve the topical application of eye drops containing antibiotics, antiviral agents, or antifungal medications [85]. However, the effectiveness of these treatments is limited due to the short retention time of the drops on the corneal surface and the low permeability of the drugs into the cornea [99,100].

**Figure 2:**
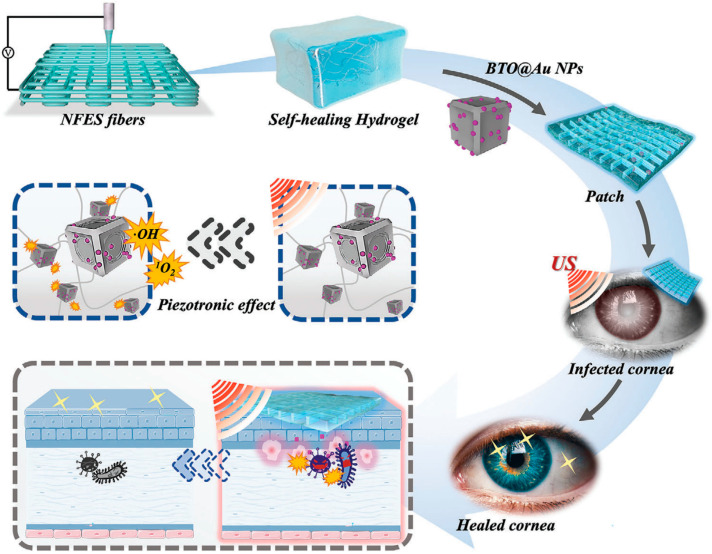
Schematic diagram illustrating the patch’s composition and functional mechanism. Reprinted with permission from ref. [98]. Copyright 2023 Wiley-VCH.

Hydrogels offer significant advantages in the treatment of keratitis. Firstly, their high water content and excellent biocompatibility make them suitable for contact with the corneal surface, providing a moist environment that promotes corneal repair [101,102]. Secondly, hydrogels can encapsulate drugs, protecting them from degradation and enabling slow, sustained release. This helps maintain therapeutic drug concentrations at the corneal site for an extended period, thereby enhancing treatment efficacy [103]. For example, Fang [66] et al. encapsulated dexamethasone into a hydrogel based on nanoemulsions and γ-cyclodextrin (DEX-NPH) for the treatment of corneal inflammation. Corneal pharmacokinetic studies demonstrated that compared to nanoemulsions, DEX-NPH containing 35% γ-cyclodextrin increased drug bioavailability by 1.29-fold, and by 4.09-fold compared to free drug solutions. In an alkali burn-induced corneal inflammation model, the hydrogel exhibited superior anti-inflammatory effects compared to nanoemulsions or free drug solutions alone. As shown in Figure 2, Kong et al. [98] developed a cornea-inspired ultrasound-responsive adhesive hydrogel patch for the treatment of keratitis. The patch is composed of recombinant human collagen (RHC) hydrogel, near-field electrospun (NFES) microfibers, and gold-nanoparticle-decorated barium titanate (BTO@Au), integrating structural biomimicry, mechanical reinforcement, tissue adhesion, and ultrasound-triggered antibacterial functions. The NFES microfibers mimic the orthogonally arranged structure of the corneal stroma, enhancing the mechanical properties of the hydrogel and guiding the aligned growth of corneal cells. The BTO@Au nanoparticles generate reactive oxygen species under ultrasound stimulation, effectively eliminating bacteria. In a rat keratitis model, the patch demonstrated superior therapeutic efficacy compared to conventional methods, offering a novel multifunctional and minimally invasive strategy for the treatment of corneal infections.

#### 3.1.2. Corneal Wound Healing

The cornea has a strong wound healing ability. However, in cases of severe trauma, infection, or corneal diseases, the normal healing process may be disrupted, leading to corneal opacity, scar formation, and vision impairment [104]. Hydrogels can play a key role in promoting corneal wound healing through various mechanisms.

**Figure 3:**
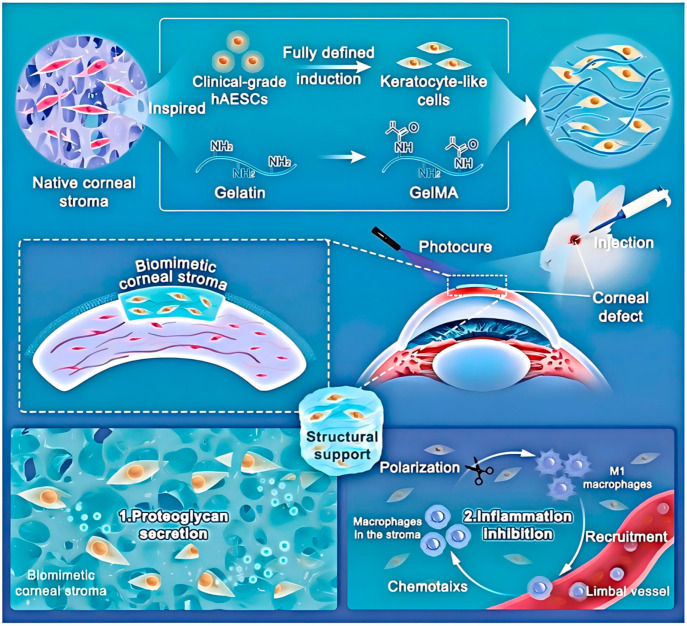
Schematic illustration of the bioengineered corneal stroma and its functional mechanisms. Guided by native stromal architecture, the construct is formed by integrating induced keratocytes with an ECM-mimicking hydrogel. Reprinted with permission from ref. [105]. Copyright 2023 Wiley-VCH.

One of the core mechanisms is to provide physical support and a moist environment for corneal cells. Hydrogels with a three-dimensional network structure can mimic the extracellular matrix of the cornea, serving as a scaffold for the adhesion, proliferation, and migration of corneal epithelial cells, stromal cells, and endothelial cells [87]. For instance, as shown in Figure 3, Huang et al. [105] developed a photocurable gelatin-based hydrogel loaded with human amniotic epithelial stem cell-derived stromal keratocytes (hAESC-SKs) for corneal wound healing. This hydrogel structure resembles the natural corneal stroma, facilitating the attachment and spreading of corneal stromal cells. Using a rabbit corneal defect model, it was demonstrated that this biomimetic corneal stroma rapidly restores corneal structure and effectively remodels the tissue microenvironment through proteoglycan secretion. It promotes transparency, suppresses the inflammatory cascade, reduces fibrosis, and synergistically decreases scar formation by approximately 75%, while also accelerating wound healing.

Hydrogels can also be loaded with bioactive factors to further accelerate corneal wound healing. Growth factors such as epidermal growth factor (EGF) and keratinocyte growth factor (KGF) are key regulators of cell proliferation, migration, and differentiation during the corneal wound healing process. Na et al. [106] strategically incorporated EGF and KGF into a hyaluronic acid (HA) hydrogel to promote corneal wound healing. The controlled release of EGF and KGF from the HA hydrogel was designed to facilitate the regeneration of both the epithelial and stromal layers. Specifically, EGF plays a critical role in the regeneration of the epithelial layer, while KGF demonstrates efficacy in the regeneration of the stromal layer. The combination of these growth factors promoted effective regeneration of each layer and demonstrated the ability to modulate each other’s regenerative effects.

#### 3.1.3. Treatment of Dry Eye Syndrome

Dry eye syndrome is a common eye condition characterized by insufficient tear production or excessively rapid tear evaporation, leading to ocular discomfort, visual impairment, and damage to the ocular surface. Traditional treatment for dry eye syndrome primarily relies on artificial tears, a type of eye drop that mimics the composition of natural tears to provide moisture and lubrication for the ocular surface. However, artificial tears have a short residence time in the eye and often require frequent application, causing inconvenience for patients [107].

**Figure 4:**
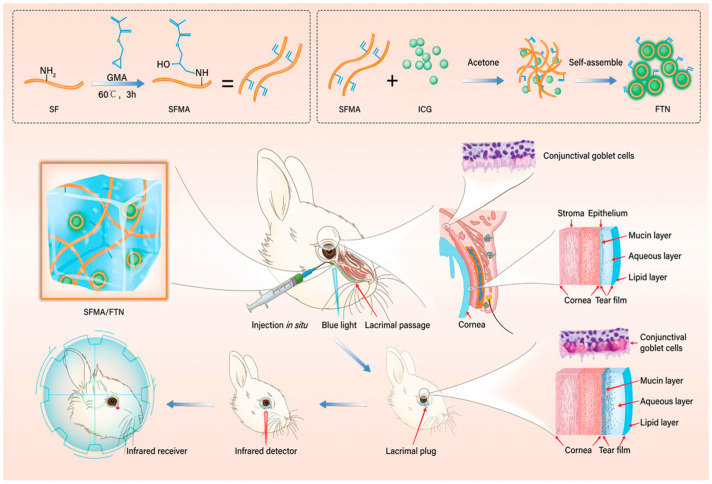
The SFMA/FTN hydrogel was synthesized and subsequently fabricated into plugs for lacrimal application. Reprinted with permission from ref. [109]. Copyright 2022 Wiley-VCH.

Hydrogels offer a more effective solution for moisturizing and lubricating the eyes in dry eye syndrome. Their high water-retention capacity helps maintain a moist environment on the ocular surface for an extended period. For instance, certain hydrogel-based eye drops or ocular inserts are designed to adhere to the surface of the cornea and conjunctiva, allowing the hydrogel to slowly release moisture and provide sustained hydration [96,108]. Ding et al. [107] developed an ion-responsive in situ gelling system targeting TRPV1, termed KVC (KAL12/V1-Cal co-assembly). Its aqueous solution rapidly forms a gel upon mixing with the ionic tear film on the ocular surface, thereby prolonging retention time while functioning as a corneal regenerative scaffold, facilitating gas exchange, and providing protection against pathogens. This hydrogel continuously releases V1-Cal to inhibit stress-activated TRPV1 channels, demonstrating excellent therapeutic efficacy in treating dry eye disease (DED). Through transcriptome sequencing and in vivo/in vitro experiments, the role of the TRPV1-Ca^2+^-pyroptosis axis in the pathogenesis of DED was confirmed, offering new insights for its treatment. Additionally, the system exhibits high biosafety and strong potential for ocular surface drug delivery with favorable therapeutic outcomes. As shown in Figure 4, Dai et al. [109] developed an in situ-forming hydrogel system based on silk fibroin as a traceable and degradable lacrimal plug for the treatment of dry eye. This system uses methacrylate-modified silk fibroin (SFMA) as the network backbone and incorporates self-assembled indocyanine green fluorescent tracer nanoparticles (FTN). The hydrogel plug is formed in situ within the lacrimal canaliculus via visible light cross-linking, achieving shape-matching with the irregular lacrimal passage. The SFMA/FTN hydrogel plug exhibits excellent biocompatibility and biodegradability, and its in vivo location and degradation process can be noninvasively monitored using near-infrared light. Experiments in a rabbit model of dry eye demonstrated that the hydrogel plug completely blocks the lacrimal duct, significantly increases tear retention, and effectively improves various clinical indicators of dry eye. This study provides a novel approach for developing absorbable lacrimal plugs with self-adaptive shaping, long-term traceability, and controllable degradation.

### 3.2. Posterior Segment Diseases

#### 3.2.1. Glaucoma

Glaucoma is a progressive optic neuropathy primarily associated with elevated intraocular pressure (IOP), which leads to irreversible optic nerve damage and visual field loss [110]. Consequently, sustained IOP reduction remains the cornerstone of current glaucoma management. Conventional therapies mainly rely on topical eye drops, including β-blockers, prostaglandin analogues, and carbonic anhydrase inhibitors. However, their short ocular residence time necessitates frequent administration, often resulting in poor long-term adherence and limited therapeutic efficacy [111,112].

To address these limitations, long-acting drug delivery strategies have been increasingly explored, including drug-eluting implants, punctal plugs, and sustained-release systems administered via the subconjunctival or intracameral routes. These approaches have significantly improved patient compliance, yet challenges remain in terms of invasiveness, reversibility, and individualized dose control. Rigid or semi-rigid drug-eluting implants can provide sustained drug release over several months but are associated with higher procedural burden and limited flexibility in dose adjustment or removal in the event of adverse reactions. In contrast, ocular surface–based inserts and punctal plugs are less invasive but generally exhibit limited penetration depth and are less suitable for targeting aqueous humor dynamics or optic nerve protection.

Hydrogel-based drug delivery systems have emerged as a versatile alternative owing to their soft, highly hydrated structure and tunable physicochemical properties [113,114,115]. Their mechanical compliance with ocular tissues enables minimally invasive administration, typically via injection, while their programmable degradation and swelling behavior offers a degree of reversibility not readily achievable with conventional implants. Importantly, hydrogels can be engineered to support sustained IOP-lowering drug release and, in some designs, to integrate additional therapeutic functions such as anti-inflammatory modulation and neuroprotective factor delivery, thereby addressing the multifactorial pathology of glaucoma.

Despite these advantages, several challenges hinder the clinical translation of hydrogel-based glaucoma therapies. Achieving consistent long-term release profiles, ensuring batch-to-batch reproducibility, and enabling scalable manufacturing remain nontrivial. In many cases, the duration of drug release achieved by hydrogels is still shorter than that provided by commercially available implants. Therefore, hydrogels are better positioned as complementary and highly customizable platforms rather than direct replacements for existing long-acting delivery systems, particularly for patient subgroups requiring precise control of release kinetics or combination therapy.

Most hydrogel-based glaucoma studies to date have been conducted in small animal models. While this limits direct clinical extrapolation, the fundamental pathological features of glaucoma—including dysregulated aqueous humor outflow, chronic optic nerve injury, and low-grade inflammation—are largely conserved across species, supporting the mechanistic relevance of these models. The localized delivery and enhanced drug utilization afforded by hydrogels also suggest potential advantages in dose reduction and treatment frequency, although rational scaling strategies will be required for human translation.

**Figure 5:**
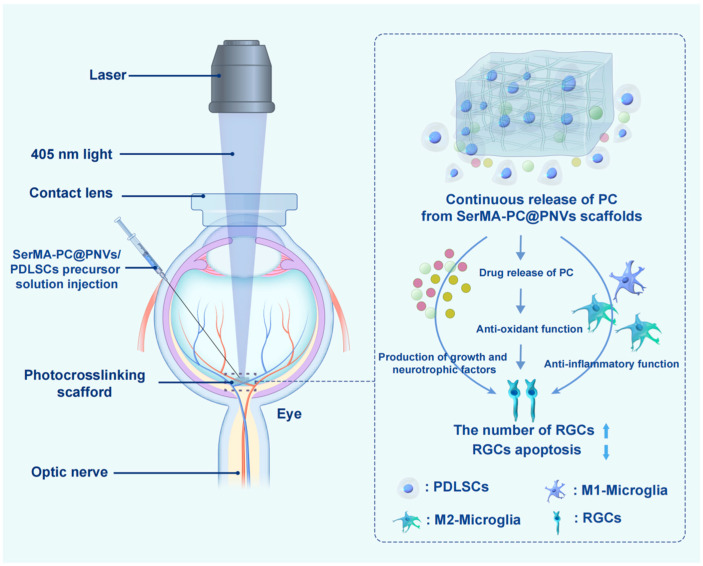
Schematic Diagram Depicting the Mechanism by Which the SerMA-PC@PNVs/PDLSCs Hydrogel Supports RGC Survival. Reprinted with permission from ref. [117]. Copyright 2026 American Chemical Society.

Beyond IOP control, neuroprotection has gained increasing attention as a complementary therapeutic goal in glaucoma [116]. Elevated IOP induces progressive retinal ganglion cell loss, underscoring the need for strategies that directly preserve neuronal integrity. In this context, hydrogel-based systems have demonstrated promise as carriers for neuroprotective agents, enabling sustained and localized delivery to the posterior segment. For example, You et al. [91] designed an injectable, drug-loaded, and reactive oxygen species-responsive multifunctional hydrogel. After intravitreal injection in a mouse model of retinal ischemia–reperfusion injury, the hydrogel continuously released exosomes and lipstatin-1 for over a month, resulting in significant restoration of visual function and reduced loss of retinal ganglion cells. The therapeutic effect surpassed that of conventional drug and exosome combinations. The findings of this study suggest that this multifunctional hydrogel holds promise for advancing retinal tissue engineering and providing innovative strategies for acute neuroprotection in retinal ischemia–reperfusion injury. As shown in Figure 5, Zhu et al. [117] developed a nanovesicle-sericin-based hydrogel scaffold (SerMA-PC@PNVs/PDLSCs) to promote the survival of retinal ganglion cells (RGCs) in glaucoma by regulating microglial polarization. The scaffold is constructed from methacrylated sericin, procyanidin-loaded nanovesicles derived from periodontal ligament stem cells (PDLSCs), and PDLSCs themselves, which are photo-crosslinked to form an in situ hydrogel. In vitro and in vivo experiments demonstrated that the hydrogel scaffold effectively reprograms microglia from a pro-inflammatory M1 phenotype to an anti-inflammatory M2 phenotype, thereby creating a neuroprotective microenvironment conducive to RGC survival. RNA sequencing analysis further revealed that the treatment significantly modulates signaling pathways related to the “inflammatory response” and “apoptotic process.” This study provides new insights into the development of neuroprotective therapies for glaucoma through immunomodulatory strategies.

Collectively, these advances highlight the potential of hydrogels to expand glaucoma therapy beyond pressure reduction alone, while emphasizing the need for systematic evaluation of long-term safety and efficacy in large animal models and early clinical studies.

#### 3.2.2. Age-Related Macular Degeneration (AMD)

AMD is a leading cause of vision impairment in older adults, primarily affecting the macula in the central part of the retina [118]. AMD is mainly classified into two types: dry AMD, characterized by the accumulation of drusen and atrophy of the retinal pigment epithelium (RPE), and wet AMD, which involves the growth of abnormal new blood vessels (choroidal neovascularization, CNV) beneath the macula. These fragile vessels are prone to leakage, releasing blood and fluid that damage the RPE and photoreceptor cells, leading to severe vision loss [119,120].

**Figure 6:**
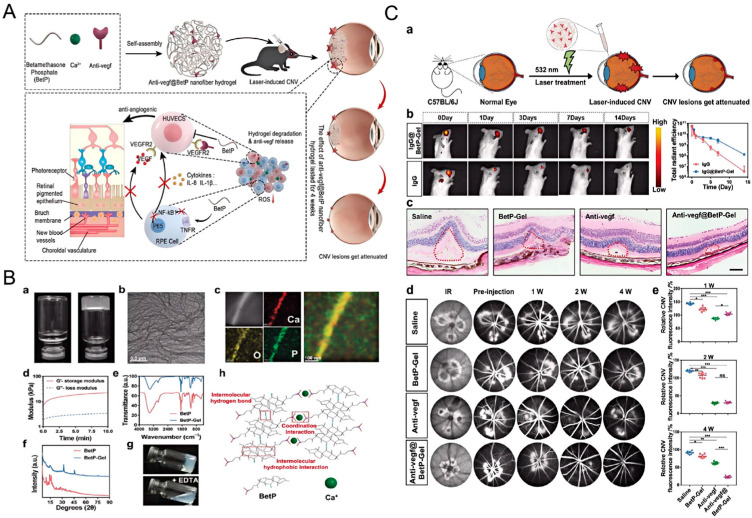
Injectable supramolecular nanofiber hydrogel with anti-inflammatory properties to enhance Anti-VEGF therapy for AMD. (**A**) Schematic representation of the fabrication of Anti-VEGF-loaded BetP nanofiber hydrogel and its therapeutic mechanism in a laser-induced choroidal neovascularization mouse model; (**B**) Characterization and gelation mechanism of the hydrogel; (**C**) Evaluation of the sustained efficacy of Anti-VEGF-loaded BetP-Gel in a laser-induced choroidal neovascularization mouse model. Reprinted with permission from ref. [119]. Copyright 2022 Wiley-VCH.

Anti-vascular endothelial growth factor (VEGF) agents are currently the standard treatment for wet AMD. However, these drugs require frequent intravitreal injections, which may lead to complications such as infection, retinal detachment, and cataract formation [121]. Hydrogels can encapsulate anti-VEGF drugs and enable sustained release, reducing the frequency of injections. For instance, as shown in Figure 6, Gao et al. [119] developed a BetP-based hydrogel (BetP-Gel) by simply combining betamethasone phosphate (BetP, a clinical anti-inflammatory drug) and anti-VEGF with CaCl_2_ for intravitreal injection. This BetP-Gel not only enables sustained long-term release of anti-VEGF to suppress retinal vascular proliferation and attenuate choroidal neovascularization, but also scavenges reactive oxygen species to reduce local inflammation. Notably, this BetP-Gel significantly extends the effective treatment duration of conventional anti-VEGF therapy. Compared with current clinical strategies that combine anti-VEGF agents with corticosteroids or non-steroidal anti-inflammatory drugs, the potential advantage of BetP-Gel does not lie in introducing entirely new therapeutic targets. Rather, it resides in the use of a hydrogel platform to achieve localized, sustained, and coordinated delivery of two distinct therapeutic signals. This mode of delivery may reduce systemic exposure and injection frequency, while also mitigating the issue of asynchronous therapeutic efficacy that often arises from pharmacokinetic mismatches in conventional combination therapies.

#### 3.2.3. Diabetic Retinopathy (DR)

DR is a microvascular complication of diabetes and a leading cause of blindness among the working-age population [122]. It is characterized by retinal vascular damage, including capillary leakage, microaneurysm formation, and neovascularization [94,123]. The pathogenesis of DR is complex, involving various factors such as hyperglycemia-induced oxidative stress, inflammatory responses, and abnormal expression of VEGF [124,125].

Hydrogels can participate in the treatment of DR through multiple mechanisms: for instance, they can deliver drugs that improve retinal microcirculation by encapsulating them within the hydrogel for slow release in the retina. Zhou et al. [14] developed a glucose-responsive hydrogel named Cu-PEI/siMyD88@GEMA-Con A (CSGC), which effectively delivers Cu-PEI/siMyD88 nanoparticles (NPs) to the retinal pigment epithelium (RPE). The Cu-PEI NPs function as antioxidant enzymes, scavenging reactive oxygen species (ROS) and inhibiting RPE pyroptosis, ultimately blocking primary blood-retinal barrier (BRB) damage by reducing microglial activation and Th1 cell differentiation. Meanwhile, the combination of MyD88 gene silencing and Cu-PEI NPs reduces IL-18 production, synergistically lowers VEGF levels, and enhances tight junction protein expression, thereby preventing secondary BRB injury. By remodeling the retinal microenvironment, the CSGC hydrogel demonstrates the potential to disrupt the vicious cycle of cross-interference between primary and secondary BRB damage, offering a promising therapeutic strategy for diabetic retinopathy (DR).

Secondly, the delivery of anti-inflammatory drugs inhibits the inflammatory response in DR. Inflammation is a critical factor in the progression of DR, and anti-inflammatory medications can reduce the production of pro-inflammatory cytokines and chemokines in the retina. Hydrogels loaded with anti-inflammatory agents such as dexamethasone enable controlled drug release, effectively suppressing retinal inflammation. In studies using DR mouse models, dexamethasone-loaded hydrogels reduced inflammatory cell infiltration in the retina and suppressed the expression of inflammatory factors, thereby delaying the progression of DR [126].

Additionally, hydrogels can be designed to deliver drugs that inhibit abnormal VEGF expression in DR. Similarly to wet AMD, excessive VEGF expression in DR leads to neovascularization. Hydrogel-encapsulated anti-VEGF drugs can block the VEGF signaling pathway, preventing the growth of abnormal retinal blood vessels [94].

Overall, by integrating multiple therapeutic strategies and enabling sustained drug release within the retina, hydrogels offer a highly promising approach for the treatment of DR.

#### 3.2.4. Retinal Detachment

Retinal detachment refers to the separation of the retina from the underlying choroid. Without timely treatment, it may lead to sudden vision loss. Traditional treatments for retinal detachment include scleral buckling, pneumatic retinopexy, and vitrectomy [127]. Hydrogels are being explored as a novel approach, serving either as vitreous substitutes or adjunctive therapy.

As a vitreous substitute, hydrogels must exhibit physical and mechanical properties similar to those of the natural vitreous, such as appropriate viscosity, transparency, and biocompatibility. Yang et al. [128] developed a self-crosslinking hydrogel composed of aldehyde-modified hyaluronic acid (HA-CHO) and amide-modified hyaluronic acid (HA-NH_2_) copolymers, which serves as a biodegradable vitreous substitute for rhegmatogenous retinal detachment (RRD) surgery. Studies demonstrated that the hydrogel could be smoothly injected into rabbit eyes via a 25-gauge needle and rapidly formed a gel, with key parameters such as light transmittance, refractive index, and modulus meeting application requirements. In vitro cytotoxicity tests confirmed its good biocompatibility without significant toxic effects. A 60-day follow-up in experimental RRD-treated rabbits revealed excellent intraocular compatibility of the hydrogel, maintenance of normal intraocular pressure (IOP), and absence of complications such as retinal redetachment or endophthalmitis.

Although current research in the posterior segment has most frequently explored hydrogels as vitreous substitutes, this perspective may underestimate their broader and more versatile potential in vitreoretinal surgery and postoperative management. In fact, compared with the complete replacement of the native vitreous, the use of hydrogels in adjunctive and functional roles may represent a more realistic and clinically translatable approach. In vitreoretinal surgery, tamponade agents are widely used to maintain retinal reattachment. Conventional silicone oil or gas tamponades are effective but are often associated with complications, including elevated intraocular pressure, emulsification, and the need for secondary removal surgery. Owing to their tunable rheological properties and compliant mechanical behavior, hydrogels hold promise as adjunctive tamponade materials, providing gentle and uniform mechanical support during the early postoperative period while avoiding the adverse effects associated with long-term rigid tamponades.

In addition, proliferative vitreoretinopathy (PVR) remains one of the leading causes of failure after retinal detachment surgery, with key pathological processes involving inflammation, cell migration, and aberrant extracellular matrix deposition. Hydrogels can serve as localized sustained-release platforms for the delivery of anti-inflammatory, antiproliferative, or antifibrotic agents, thereby suppressing the onset and progression of PVR during critical postoperative windows [79,129]. Compared with systemic administration or repeated intravitreal injections, this strategy is more conducive to achieving sustained, localized, and controllable therapeutic effects. Therefore, from a clinical perspective, the value of hydrogels in posterior segment diseases extends beyond the ultimate goal of “vitreous substitution.” Their multifunctional roles as adjunctive tamponade materials, postoperative drug delivery systems, and microenvironment-modulating platforms may lower the barriers to clinical adoption and provide a more pragmatic translational pathway for hydrogels in vitreoretinal disorders.

## 4. Drug Delivery Mechanisms of Hydrogels in Ophthalmology

### 4.1. Passive Diffusion

Passive diffusion is the fundamental mechanism for ocular drug delivery via hydrogels. Its core lies in the natural migration of drug molecules from the interior of the drug-loaded hydrogel (high-concentration region) to the surrounding ocular tissues (low-concentration region), relying on the concentration gradient after the hydrogel contacts the ocular environment without the need for external energy input. The diffusion rate is mainly regulated by three factors: small-molecule drugs have better ability to penetrate the hydrogel network than macromolecular drugs, resulting in faster diffusion; the pore size of the hydrogel increases with the decrease in crosslinking density, and larger pore size leads to smaller drug diffusion resistance; the swelling of the hydrogel can expand the network pore size and improve drug fluidity, and high swelling degree can accelerate drug release [130].

However, this mechanism has inherent limitations: the diffusion process is completely dependent on the concentration gradient. As the concentration difference in drug release decreases, the drug release rate decreases significantly in the later stage, making it difficult to maintain a sustained therapeutic concentration. In addition, it lacks targeting, and drugs are prone to diffuse to normal ocular tissues, increasing the risk of toxic and side effects. At the same time, the corneal penetration efficiency of drugs is typically less than 5%, further affecting the drug delivery efficiency, which limits its application in the treatment of chronic and refractory ocular diseases [131,132,133].

### 4.2. Responsive Release

Responsive release is the core mechanism allowing hydrogels to achieve precise ocular drug delivery. By sensing specific microenvironmental changes (e.g., temperature, pH value) at ocular lesion sites or external stimuli, hydrogels trigger the controlled release of drugs, which can precisely match the progression of pathological changes, enhance therapeutic efficacy, and reduce side effects on the systemic circulation and normal ocular tissues. This mechanism is particularly suitable for diseases with characteristic microenvironmental changes, such as infectious and inflammatory ocular diseases.

#### 4.2.1. Temperature-Responsive Release

Temperature-responsive release is a key strategy for intelligent ocular drug delivery, relying on thermosensitive hydrogels that undergo temperature-dependent structural changes to achieve drug release. Local temperature elevations at ocular lesion sites (e.g., inflammatory and infectious foci) can act as endogenous trigger signals. Exogenous near-infrared photothermal heating can also be used to regulate drug release, enabling on-demand drug delivery, increasing local drug concentrations at lesion sites, and reducing drug exposure to normal ocular tissues [83].

However, this drug delivery system still faces significant challenges. The narrow temperature range of the normal eye and the limited temperature fluctuation amplitude at lesion sites restrict the response sensitivity of hydrogels. Exogenous excessively high temperatures can easily damage delicate ocular tissues such as the cornea and retina, imposing stringent requirements on the response threshold and temperature control precision of hydrogels [134]. Current research focuses on optimizing thermosensitive polymers to narrow the phase transition temperature range and improve biocompatibility, thereby balancing drug release efficiency and tissue safety to facilitate clinical translation.

#### 4.2.2. pH-Responsive Release

pH-responsive hydrogels can react to changes in the pH of the ocular environment. These hydrogels typically contain acidic or alkaline functional groups such as carboxyl groups (-COOH) and amino groups (-NH_2_). When the pH of the surrounding environment changes, the ionization degree of these functional groups is altered, thereby affecting the swelling capacity and drug release behavior of the hydrogel. The pH value of the normal physiological environment of the eye ranges from 7.0 to 7.4, while ocular diseases such as infectious and inflammatory disorders can induce a significant deviation in the pH value of the local tissue microenvironment. For instance, Zhang [84] et al. integrated sericin with oxidized chitosan nanoparticles loaded with black phosphorus quantum dots (BPQDs) via Schiff base chemistry to fabricate a functional pH-responsive hydrogel. After corneal injury, the hydrogel selectively releases BPQDs in response to the acidic microenvironment, thereby inhibiting the innate immune cascade-driven fibrosis mediated by the PMN-ROS-NET axis.

Despite their applications in ophthalmology, pH-responsive hydrogels still have limitations, including insufficient response sensitivity caused by the small amplitude of pathological pH deviation in some diseases, interference of the ocular surface microenvironment with response precision, great difficulty in regulating the mechanical properties and swelling rate of materials, and bottlenecks in clinical translation such as the lack of large-sample clinical validation data.

## 5. Research Status and Existing Problems of Hydrogels in the Treatment of Ophthalmic Diseases

### 5.1. Current Research Findings

In recent years, significant progress has been made in the research of hydrogels for the treatment of ophthalmic diseases, particularly in the development of novel hydrogel materials and the optimization of drug delivery systems.

In the field of novel hydrogel materials, researchers have been continuously exploring various natural and synthetic polymers to develop hydrogels with improved properties. For instance, natural polymers such as hyaluronic acid and chitosan have been further modified to enhance their performance—hyaluronic acid-based hydrogels have been engineered to exhibit better mechanical strength and more sustained drug release capabilities [135,136,137]. Innovations have also been made in the design of synthetic polymers. Some researchers have developed biodegradable synthetic hydrogels with precisely controllable degradation rates. These hydrogels can release drugs at specific rates and gradually degrade within the eye, eliminating the need for removal after treatment [138,139,140].

Notable achievements have also been made in optimizing drug delivery systems. Multi-stimuli-responsive hydrogels with multifunctional properties have been successfully developed. Yu et al. [141] constructed a three-dimensional cross-linked hydrogel system using carboxymethyl chitosan (CMC) and poloxamer F127 (PEO-PPO-PEO block copolymer), which exhibits reversible sol–gel transition in response to temperature/pH at low concentrations. Characterization revealed optimal pore size and swelling properties at 35 °C and pH 7.4, with a gelation temperature of 32–33 °C. The hydrogel showed no cytotoxicity at low concentrations and demonstrated sustained release of nepafenac, with the fastest release observed at 35 °C and pH 7.4, indicating its suitability for ophthalmic drug delivery.

The integration of hydrogels with nanotechnology has also opened new pathways for drug delivery. Hydrogels loaded with nanoparticles can enhance drug loading capacity and release performance, not only protecting the drugs from degradation but also improving their penetration into targeted ocular tissues [142,143,144].

### 5.2. Challenges

#### 5.2.1. Stability and Shelf Life

Hydrogels face significant challenges in terms of stability and shelf life when applied in the treatment of ophthalmic diseases [144,145,146]. During storage, they are prone to degradation, dehydration (drying), and drug leakage. For hydrogels made from biodegradable polymers such as PLGA, degradation reactions like backbone hydrolysis may occur, leading to a reduction in crosslinking density and polymer chain breakage [139]. This can result in premature drug release or fluctuations in the drug release rate. Their high hydrophilicity makes them susceptible to water loss and contraction under improper storage conditions, altering physical properties such as swelling behavior and mechanical strength. This not only affects adhesion to the ocular surface and moisturizing efficacy but also influences the drug diffusion rate due to changes in pore size. Additionally, defects in the hydrogel network or long-term weakening of the interaction between the drug and the matrix may cause drug leakage, which reduces the initial effective drug loading and compromises therapeutic efficacy, while also increasing the risk of side effects if the initial drug loading is increased.

#### 5.2.2. Safety and Long-Term Risk Considerations of Intravitreal Hydrogel Injection

Compared with ocular surface or subconjunctival delivery, the intravitreal environment imposes substantially more stringent requirements on the safety and stability of injectable materials [147]. Vitreous transparency is a critical parameter for evaluating the feasibility of intraocular hydrogels. Any microphase separation, particle aggregation, or refractive index mismatch occurring during gelation, swelling, or degradation may induce light scattering and compromise visual quality. Consequently, hydrogels intended for vitreous applications are generally required to exhibit a highly homogeneous network structure, a refractive index close to that of the native vitreous, and the ability to maintain optical transparency over extended periods in vivo.

Intraocular inflammatory responses represent another major risk limiting the clinical translation of injectable hydrogels [148]. Even when the bulk material demonstrates favorable biocompatibility, residual crosslinkers, unreacted monomers, or low-molecular-weight degradation products may activate microglia or recruit inflammatory cells, thereby triggering chronic, low-grade inflammation. Previous studies suggest that reducing crosslinking density, employing natural or biomimetic polymers, and avoiding highly reactive crosslinking chemistries can mitigate intraocular inflammatory responses to some extent. However, these strategies require systematic validation in long-term experimental models.

The degradation behavior of hydrogels and the safety of their by-products are of particular importance in the context of chronic ocular diseases [149]. Conditions such as glaucoma and retinal disorders often necessitate long-term or repeated treatment, making the degradation rate, degradation pathway, and potential accumulation of degradation products within the vitreous critical considerations. Ideally, intraocular hydrogels should degrade in a predictable and gradual manner after fulfilling their drug delivery or structural support functions, yielding non-toxic and metabolizable small molecules without disrupting aqueous humor dynamics or retinal function.

Overall, while injectable hydrogels offer distinct advantages for minimally invasive, long-acting intraocular therapy, their safety in vitreous applications must be established through comprehensive and long-term evaluations of intraocular biocompatibility and visual function. Addressing these issues represents an unavoidable and essential challenge for future research and clinical translation.

#### 5.2.3. Large-Scale Production Capacity and Cost Efficiency

The scalability and cost-effectiveness of hydrogel production are key factors limiting their widespread clinical application [150]. Scaling up the production of high-quality, consistent hydrogels remains challenging, particularly for hydrogels involving complex crosslinking reactions or multiple components. Their manufacturing processes are difficult to scale up—for instance, chemical crosslinking methods require precise control of temperature, pH, and reactant concentration to ensure uniform product performance. However, in large-scale production, it is challenging to accurately control reaction conditions in large-volume reactions, which can easily lead to batch-to-batch variations in properties such as swelling behavior, drug loading capacity, and mechanical strength, thereby resulting in inconsistent clinical efficacy. Additionally, some polymers used in hydrogels (such as well-defined synthetic polymers with low immunogenicity and high-purity natural polymers) are expensive, and the inclusion of additives like crosslinking agents, drugs, and nanoparticles further increases costs, driving up overall production expenses. Moreover, certain hydrogels (such as multi-stimuli-responsive smart hydrogels) require specialized equipment, multi-step processing, and skilled personnel for preparation. The complexity of these manufacturing processes further raises production costs. Together, these factors constitute major obstacles to the commercial development and widespread application of hydrogel-based ophthalmic treatments, particularly in regions with limited medical resources.

#### 5.2.4. Standardization and Regulatory Issues

The standardization and regulatory framework for hydrogels as ophthalmic drug carriers are still being refined, facing numerous challenges [151,152]. Currently, there is a lack of comprehensive and unified quality control standards for ophthalmic hydrogels. Evaluation methods for properties such as biocompatibility, drug release rate, and mechanical strength vary across research teams and manufacturers, making it difficult to compare the performance of different products and ensuring consistent production quality (e.g., deviations in drug release rate measurements due to differences in release media, experimental temperature, agitation conditions, and drug quantification techniques can affect the accuracy of efficacy assessments). As a relatively novel drug delivery system, regulatory agencies have yet to establish clear and unified approval guidelines. Moreover, the unique interactions between hydrogels and ocular tissues necessitate tailored in vitro/in vivo models for long-term safety (e.g., chronic inflammation, immune responses, and risks associated with the accumulation of degradation products) and efficacy evaluations, which are still under development, creating uncertainties for researchers and manufacturers. Additionally, clinical trial design poses challenges. Since the drug release profiles and ocular interactions of hydrogels differ from those of traditional drugs, conventional clinical trial designs cannot be directly applied. Factors like control group selection, optimal dosage and administration frequency, and efficacy evaluation endpoints require careful consideration. Furthermore, long-term follow-ups needed to assess the durability of efficacy and delayed adverse effects further increase the complexity and cost of clinical trials.

## 6. Future Prospects and Development Trends

### 6.1. Development of New Hydrogel Materials

The future development of ophthalmic gel materials holds significant potential. First, the focus is on developing materials with superior biocompatibility. Researchers are exploring novel polymers and modification methods, delving into the molecular-level interaction mechanisms between hydrogels and ocular tissues. By designing hydrogels that closely mimic the natural components of the eye, the risks of immune reactions and cytotoxicity can be further reduced, creating a suitable microenvironment for ocular cell survival and function while minimizing adverse reactions [153,154]. Second, efforts are being made to enhance the drug-loading capacity of hydrogels. Novel hydrogels with unique structures, such as hierarchically porous hydrogels or those containing specific drug-binding sites, are being developed. The hierarchical porous structure increases drug encapsulation space and regulates drug diffusion and release, while hydrogels with specific binding sites improve loading efficiency through strong interactions like covalent bonds or specific molecular recognition [81,155]. This enables high drug-loading capacity and stable encapsulation, meeting the demands of high-dose drug administration for severe ocular diseases. Third, there is a push to develop hydrogels with more precise responsive characteristics. Beyond existing temperature-, pH-, and light-responsive types, researchers are working on hydrogels that can respond to multiple stimuli simultaneously or sequentially, such as temperature changes due to ocular inflammation and disease-specific biomarkers. By integrating multiple sensitive components into the structure and precisely controlling the response mechanisms, targeted drug release at the lesion site can be achieved. Additionally, the development of self-healing hydrogels can repair damage caused by external forces or degradation, enhancing their stability and durability in the complex ocular environment and ensuring sustained and stable drug release.

### 6.2. Hydrogel Combination Therapy

The combination of hydrogels with other therapeutic approaches demonstrates significant potential in the treatment of ophthalmic diseases. In the field of phototherapy, taking age-related macular degeneration (AMD) as an example, hydrogels can encapsulate photosensitizers and deliver them stably to targeted ocular sites. Under specific wavelengths of light, the photosensitizers generate reactive oxygen species, selectively destroying abnormal choroidal blood vessels and inhibiting neovascularization. Simultaneously, the hydrogels protect the photosensitizers from degradation and reduce the side effects associated with conventional phototherapy by precisely controlling their release and activation [156].

In the context of gene therapy for inherited ocular diseases, such as certain forms of retinitis pigmentosa, hydrogels can serve as auxiliary carrier platforms for gene delivery systems, enabling localized retention and controlled release of genetic vectors [157]. For non-viral vectors, which are often limited by relatively low transfection efficiency and short-lived gene expression, hydrogels may enhance the probability of uptake by retinal target cells by regulating local vector concentration and the temporal delivery window, thereby potentially reducing dosing frequency. However, the therapeutic benefit of this strategy is highly dependent on the biological characteristics of the vector and the design of the release kinetics. Rather than simply prolonging release duration, the primary advantages of hydrogels in gene therapy lie in spatial confinement, attenuation of the initial exposure peak, and improved local biocompatibility, which together may help reduce off-target transfection and immune-related adverse responses, ultimately contributing to improved treatment safety.

In the emerging field of cell-based therapy, particularly for the treatment of corneal diseases, hydrogels can function as platforms for cell delivery and microenvironment modulation by encapsulating corneal epithelial cells or stem cells and enabling their localized retention [158]. By providing a three-dimensional, extracellular matrix–like structure, hydrogels can support early cell adhesion and survival and, to a certain extent, buffer the adverse effects of post-transplant hypoxia and limited nutrient availability, thereby creating a relatively favorable microenvironment for corneal repair. However, the long-term therapeutic outcome remains highly dependent on the hydrogel’s capacity to support oxygen diffusion and cellular metabolism, regulate immune responses, and facilitate effective integration between transplanted cells and host tissue. Collectively, these factors will determine whether corneal regeneration can progress beyond structural repair toward stable functional restoration.

In the treatment of retinal diseases, hydrogels can deliver therapeutic cells, such as retinal progenitor cells, to targeted sites, protecting the cells during transportation and implantation. Moreover, through controlled release, they enhance the integration of cells with damaged retinal tissues, improving therapeutic outcomes.

## 7. Conclusions

Hydrogels, owing to their excellent physicochemical properties and biocompatibility, have emerged as core materials with great translational potential in the field of ophthalmic therapy. Their translational process from the laboratory to the clinic is reshaping diagnosis and treatment models and effectively addressing clinical pain points. Currently, translational applications have covered key areas such as corneal diseases, glaucoma, retinal diseases, and dry eye syndrome. The drug delivery mechanisms of hydrogels serve as the core support for successful translation; these mechanisms precisely regulate the drug release rate and targeting ability, breaking through the bottlenecks of “low bioavailability and severe side effects” associated with traditional ophthalmic drugs and further enhancing the value of clinical translation.

However, the clinical translation of hydrogels in ophthalmology still faces three major bottlenecks: first, insufficient storage stability, which makes it difficult to meet supply chain requirements; second, great difficulty and high cost in large-scale production, which limit their popularization at the grassroots level; third, the lack of unified quality standards and unclear regulatory processes, which prolong the translation cycle. Future translation efforts should be guided by “addressing clinical pain points and improving efficiency”. Research and development should focus on breaking through key indicators in line with translational demands, while exploring the combined application of hydrogels with phototherapy, gene therapy, and other modalities to expand the therapeutic boundaries for refractory ocular diseases. Additionally, it is imperative to promote the establishment of standardization and the simplification of approval processes to provide institutional guarantees for translation.

In general, the translational impact of hydrogels on ophthalmic therapy is revolutionary. They represent not only material innovation but also a reconstruction of diagnosis and treatment models. After overcoming translational bottlenecks and exploring new directions, hydrogels will accelerate their transformation from laboratory achievements into routine clinical tools, providing patients with higher-quality personalized treatment. This will drive the upgrading of ophthalmic therapy toward precision, long-term efficacy, and minimal invasiveness, ultimately improving patients’ visual prognosis and quality of life.

## Figures and Tables

**Table 1:** Summary of some drug/active ingredient-loaded hydrogels applied in the treatment of ophthalmic diseases in recent years.

Disease	Hydrogel	Drug/Active Ingredient	Key Results	Ref.
Corneal inflammation	Nanoemulsion-based pseudopolyrotaxane hydrogel	Dexamethasone	Drug availability increased by 4.09 times; effective treatment for corneal inflammation	[66]
Fungal keratitis	Guanosine supramolecular hydrogel	Biogenic cinnamaldehyde, tannic acids	Good therapeutic effect on mice with Candida keratitis	[85]
Bacterial keratitis	Poly-PEG/PPG urethane hydrogel	Erythromycin	A potent therapeutic effect in a mouse model of bacterial keratitis	[86]
Bacterial keratitis	Sodium alginate and gelatin	CuS/MnS nanocomposites Self-assembled diphenylalanine dipeptide	Killing bacteria, reducing inflammation, and promoting wound healing Good biocompatibility with human corneal epithelial cells	[74]
Corneal wound healing	Aldehyde-modified oxidized guar gum (OGG) and carboxymethyl chitosan	Mesenchymal stem cell exosomes	Significantly improving corneal wound repair by promoting collagen deposition and reducing inflammation	[87]
Corneal wound healing	Gelatin methacryloyl	Mesenchymal stem cell	Reducing inflammation, promoting the repair of corneal epithelium and limbus, and minimizing scar formation in the stroma	[88]
Corneal wound healing	4XT recombinant protein Four-arm polyethylene glycol succinimidyl glutarate	TGF-β1 siRNA and cerium oxide nanoparticles	Achieving scarless healing of corneal wounds	[89]
Glaucoma	PEG-PLA	Latanoprost and timolol	The duration of intraocular pressure reduction exceeds 28 days, with a relative pharmacological availability (PA) 5.7 times greater than that of eye drops	[90]
Glaucoma	Quaternary ammonium chitosan (QCS)/ tannic acid	Exosomes/Liproxstatin-1	Significantly ameliorating the damage to retinal ganglion cells	[91]
Glaucoma	Poly(trimethylene carbonate)15–F127–poly(trimethylene carbonate)15	Mitomycin C	Good control of intraocular pressure Inhibition of scar formation	[92]
Retinal diseases	Carboxy methyl chitosan and oxidized dextran	iPSC-derived choroidal endothelial cell(CEC)	When transplanted into hydrogel, the retention and survival of iPSC-derived CECs are significantly enhanced compared to being in a single-cell suspension.	[11]
Diabetic retinopathy	hyaluronic acid methacryloyl	Aflibercept and miR-21-3p antagomir	Effectively inhibiting VEGF-induced vascular dysfunction	[93]
Diabetic retinopathy	Agarose	siRNA targeting Cx43 nanoparticles and anti-VEGF	Reducing angiogenesis, inflammation, and apoptosis	[94]
Rhegmatogenous retinal detachment	Gelatin methacryloyl	Glucose	Retinal reattachment was successful in 75% of the cases, significantly higher than the 16.7% reattachment rate in the control group	[12]
Dry eye disease	Silk fibroin nanoparticle hydrogel	NK1R antagonist	Maintaining a stable CP concentration for 25 h	[95]
Dry eye disease	Oxidized HA-containing aldehyde groups and gelation	Polyethylene imine-functionalized carbon dots	Reducing oxidative damage and suppressing the expression of inflammatory factors	[96]

## Data Availability

No new data were created or analyzed in this study.
